# A Case Study of Severe Esophageal Dysmotility following Laparoscopic Sleeve Gastrectomy

**DOI:** 10.1155/2016/9363545

**Published:** 2016-06-19

**Authors:** Caroline E. Sheppard, Daniel C. Sadowski, Richdeep Gill, Daniel W. Birch

**Affiliations:** ^1^Centre for the Advancement of Minimally Invasive Surgery, Royal Alexandra Hospital, University of Alberta, Rm 511 CSC, 10240 Kingsway Avenue, Edmonton, AB, Canada T6X 1R8; ^2^Department of Medicine, Division of Gastroenterology, Zeidler Ledcor Centre, University of Alberta, Edmonton, AB, Canada T6G 2X8; ^3^Rm 3656 West Wing, Peter Lougheed Hospital, 3500 26th Avenue NE, Calgary, AB, Canada T1Y 6J4

## Abstract

Following bariatric surgery, a proportion of patients have been observed to experience reflux, dysphagia, and/or odynophagia. The etiology of this constellation of symptoms has not been systematically studied to date. This case describes a 36-year-old female with severe esophageal dysmotility following LSG. Many treatments had been used over a course of 3 years, and while calcium channel blockers reversed the esophageal dysmotility seen on manometry, significant symptoms of dysphagia persisted. Subsequently, the patient underwent a gastric bypass, which seemed to partially relieve her symptoms. Her dysphagia was no longer considered to be associated with a structural cause but attributed to a “sleeve dysmotility syndrome.” Considering the difficulties with managing sleeve dysmotility syndrome, it is reasonable to consider the need for preoperative testing. The question is whether motility studies should be required for all patients planning to undergo a LSG to rule out preexisting esophageal dysmotility and whether conversion to gastric bypass is the preferred method for managing esophageal dysmotility after LSG.

## 1. Introduction

Laparoscopic sleeve gastrectomy (LSG) is a commonly performed bariatric surgical procedure. LSG involves removal of approximately 75% of the stomach, leaving a narrow tubular stomach, similar in diameter to the esophagus. Following bariatric surgery, a proportion of patients have been observed to experience reflux, dysphagia, and/or odynophagia. The etiology of this constellation of symptoms has not been systematically studied to date. Often these symptoms are treated empirically with proton-pump inhibitors or dilation of strictures despite the lack of evidence for acid-peptic pathology or mechanical obstruction [1]. We present a case of severe esophageal dysmotility following LSG.

## 2. Case

A 36-year-old female with a BMI of 39.7 kg/m^2^ underwent an uncomplicated laparoscopic sleeve gastrectomy using a 50 F bougie with dissection 6 cm proximal to the pylorus. Her prior medical history consisted of pulmonary embolism (PE), neurocardiogenic syncope, and back pain. She denied any symptoms of dysphagia or gastroesophageal reflux preoperatively. Three months after LSG, she developed recurrent mild retrosternal pain. Her imaging was negative for a PE, and she was treated with proton-pump inhibitors for presumed gastroesophageal reflux. She underwent gastroscopy, CT, and full cardiac work-up, which were unremarkable. No hiatal hernia, stricture, ulcer, leak, partial dilation of the sleeve, retained fundus, or abnormality in the gastroesophageal junction was observed. However, over the next 6 months the symptoms worsened, and she presented to hospital 8 times requiring admission for assessment of severe high epigastric pain.

One year after LSG, esophageal manometry and 24 h pH studies were performed to investigate a possible esophageal etiology of her pain. The manometry study demonstrated a pattern consistent with hypertensive peristalsis with an average distal contractile interval (DCI) of 5216 mmHg/sec/cm (normal DCI = 500–5000) with solicited swallows. The 24 h esophageal pH study was normal (DeMeester score of 15.0) with a negative Symptom Index score (0.0%) between acid reflux episodes and chest pain symptoms. Her symptoms of dysphagia continued, and she steadily declined in weight to a BMI of 27.8. To treat the hypertensive peristalsis, the patient was begun on therapy with diltiazem 30 mg QD.

Because of continuing symptoms while on diltiazem, further investigations were carried out one year later. A second manometry demonstrated weak lower esophageal sphincter pressure, with normalization of manometry parameters while on diltiazem (Table 1). An esophageal 24 h impedance study was normal (DeMeester score of 3.2). During the study a high number of nonacid reflux episodes occurred (*n* = 71), but this was not significantly linked to Symptom Association Probability (74%). She continued to have severe retrosternal chest pain and episodes of dysphagia with solids, despite evidence that the hypertensive peristalsis appeared to have improved with therapy. Botox injection of 100 units at the gastroesophageal junction was performed in order to attempt relieving the esophageal spasms. These appeared to have little effect on the patient's symptoms.

Dysphagia symptoms began to worsen to both liquids and solids, and multiple emergency room visits were again observed. More than 3 years after LSG, various treatments had been used to treat her esophageal spasms, including calcium channel blockers (diltiazem), LABA-2 (Symbicort), vasodilators (Nitrate), antispasmodic medication (Lyrica, Gabapentin, and Botox), analgesics (Tylenol 4, Tramadol, Butrans, oxyNEO, viscous lidocaine, Hydromorph contin, Fentanyl, Methadone, Dilaudid, Morphine, and Clonidine), muscle relaxants (Baclofen, Tizanidine, Zanaflex, and Cyclobenzaprine), antimigraine (Zomig), promotility (Domperidone), antiemetic medication (Zofran), antireflux medication (Nexium, Omeprazole, and Pantoloc), benzodiazepine (Ativan), nonbenzodiazepine hypnotics (Zopiclone), cannabinoid (Cesamet), tricyclic antidepressant (Nortriptyline, Elavil), serotonin norepinephrine reuptake inhibitor (Cymbalta), and selective serotonin reuptake inhibitors (Prozac). Proposed treatment options for this escalating esophageal pain included Botox injection to the pylorus, pyloromyotomy, partial esophageal myotomy, or a gastric bypass to try and reduce the hypothesized high-pressure sleeve. As a last resort, some surgeons may also consider a total gastrectomy. After discussion with the patient, a laparoscopic Roux-en-Y gastric bypass was performed, which seemed to relieve the dysphagia and retrosternal pain.

After the gastric bypass, ER and outpatient visits both decreased twofold (0.5 versus 0.2 ER visits/month and 0.6 versus 0.3 outpatient visits/month) attributed to pain relief. Presently 5 years following LSG, pain symptoms are being managed with analgesics and neuropathic treatment is being considered. This complicated patient has had over 100 visits with specialists over the past 6 years to manage her obesity and chronic dysphagia. Her dysphagia is no longer considered to be associated with a structural cause but is now attributed to a “sleeve dysmotility syndrome.”

## 3. Discussion

Esophageal dysmotility occurs when the muscles and sphincters of the esophagus have impaired coordination, altered contraction strength, and/or contractile duration causing impaired esophageal transit. The combination of these abnormalities after LSG has not yet been described.

Symptoms of foregut dysmotility are disconcerting when they arise following LSG. These symptoms are varied and include dysphagia, odynophagia, nausea, vomiting, heartburn, and pain.

Carabotti et al. found that dysphagia developed in 19.7% of patients after LSG, which manifested in retrosternal or throat discomfort when consuming solids or liquids [2]. A significant increase in dyspepsia (59.4%) was also attributed to increased pressure in the sleeve [2]. Kleidi et al. found a combination of reflux and dysphagia significantly increased after LSG [3]. Additional reports describe dysmotility after the laparoscopic adjustable gastric band as causing symptoms of dysphasia and reflux [4]. These symptoms normally resolve after adjustment or removal of the band. In contrast, dysmotility following LSG may be irreversible.

Our case demonstrated manometric evidence for hypertensive peristalsis. It is unclear if this disorder was present before LSG surgery, whether this was a preexisting condition that was exacerbated by the LSG, or whether the syndrome was created by the LSG. However, treatment with calcium channel blockers reversed the manometric abnormalities but failed to resolve symptoms. Sleeve dysmotility syndrome causes persistent dysphagia and reflux-like symptoms and may respond partially to gastric bypass.

It is difficult to determine whether technique contributes to this sleeve dysmotility syndrome, as many of these esophageal syndromes are idiopathic. Bougie size for LSG and its impact on leak rate and gastroesophageal reflux have been greatly discussed in the literature. Parikh et al. described in their meta-analysis using data from nearly 10,000 patients that a bougie size equal or greater to 40 F decreased the odds of developing a postoperative leak [5]. The literature on technique contributing to gastroesophageal reflux symptoms has many theories (i.e., retained fundus, blunted angle of His, bougie size, resection of antrum, high-pressure system, etc.). This patient was negative for both leak and acid reflux, which made it challenging to assess whether technique contributed to the patient's symptoms based on current literature. The patient had manometric abnormalities, and the causal relationship of LSG technique and esophageal dysmotility has yet to be defined.

The LSG has been described as creating a high-pressure system in the sleeve from simultaneous gastric and pyloric contractions [6]. When filled with saline, the intragastric pressure is increased after LSG (43 mmHg) compared to normal gastric anatomy (34 mmHg) [7]. By reducing the “high-pressure” system to a “low-pressure” system, that is, by gastric bypass, our hope was that this would alleviate the hypertensive esophagus and esophageal spasms. The gastric bypass has been successful for improving or resolving other gastroesophageal issues after the LSG, such as uncontrollable gastroesophageal reflux [8], and may be the preferential choice for managing dysmotility.

Preoperative manometry is used to avoid major postoperative issues of dysphagia before antireflux surgery. Concurrent 24 h pH testing is also used to confirm the presence of reflux. These results can detect an upper range of 1 of 14 patients being inappropriate for surgical intervention [9]. Consequently, preoperative manometry may be a method to screen patients with dysmotility in order to select an appropriate bariatric procedure. This would avoid significant postoperative complications and the ultimate need for reoperation.

This is a complicated question that has significant impact on the investigation burden placed on the patient. Considering the difficulties with managing sleeve dysmotility syndrome, it is reasonable to consider the need for preoperative testing. The question is whether motility studies should be required for all patients planning to undergo a LSG. Manometry results would identify patients that may not be able to tolerate a high-pressure sleeve from either esophageal spasms, hypertensive esophagus, achalasia, or scleroderma. Consequently, they may be better candidates for a gastric bypass.

## Figures and Tables

**Table 1:** Changes in esophageal motility after 30 mg diltiazem QD therapy.

Esophageal manometry measurement	Before diltiazem	After diltiazem	Normal value
Completed peristalsis (%)	100	100	≥80%
LES pressure (mmHg)	40.5	10.8	13.0–43.0
LES residual pressure (mmHg)	13.4	4.0	<15.0
Contraction amplitude (mmHg)	210.4	76.6	30.0–180.0
High amplitude contraction (%)	100.0	0.0	—
Distal contractile integral (mmHg/s/cm)	5216.4	1481.7	500.0–5000.0
